# Congenital Hyperinsulinism in the Era of Precision Medicine: Genetically Driven Diagnosis and Novel Therapies

**DOI:** 10.3390/children13101431

**Published:** 2026-10-18

**Authors:** Jaikumar B. Contractor, Lalankumar Bharti, Ishan Shah, Stuti Shah, Somashekhar Nimbalkar, Anurag Bajpai, Ravindra Kumar, Viswanathan Mohan, Venkatesan Radha, Pratik Shah

**Affiliations:** 1 Department of Anatomy, GMERS Medical College, Panchmahal, Godhra 389120, Gujarat, India; 2 Congenital Hyperinsulinism India Association, Anand 388325, Gujarat, India; info@hyperinsulinism-india.org or ishan5jan@gmail.com (I.S.); 2219361020136@paruluniversity.ac.in or stutiashishshah@gmail.com (S.S.); somu_somu@yahoo.com or somashekhar.pediatrics@aiimsdeoghar.edu.in (S.N.); 3 Jag Pravesh Chandra Hospital, Government of Delhi, New Delhi 110053, India; pediatricsjpch25@gmail.com or drlkbharti2011@gmail.com; 4 Parul Institute of Medical Sciences & Research, Vadodara 391760, Gujarat, India; 5 All India Institute of Medical Sciences, Deoghar 814117, Jharkhand, India; 6 Regency Center for Diabetes, Endocrinology & Research (CDER), Kanpur 208001, Uttar Pradesh, India; anurag.bajpai@regencyhealthcare.in or dranurag.bajpai@gmail.com; 7 Hindu Rao Hospital & North Delhi Municipal Corporation Medical College, Delhi 110007, India; ravindra1kumar1@gmail.com; 8 Madras Diabetes Research Centre, ICMR Collaborating Centre of Excellence, Chennai 600086, Tamil Nadu, India; drmohans@diabetes.ind.in (V.M.); radharv@yahoo.co.in (V.R.); 9 Dr. Mohan’s Diabetes Specialities Centre, IDF Centre of Excellence in Diabetes Care, Chennai 600086, Tamil Nadu, India; 10 The Royal London Childrens Hospital, Barts Health NHS Trust, E1 1FR & Queen Mary University of London, London EC1M 6BQ, UK

**Keywords:** genotype–phenotype correlation, K_ATP_ channel mutations, diazoxide-unresponsive hypoglyacemia, ^18^F-DOPA PET/CT, hyperinsulinism

## Abstract

**Highlights:**

**What are the main findings?**

- The expanding genetic spectrum of congenital hyperinsulinism includes defects in K_ATP_ channels, metabolic pathways, transcription factors, membrane transport, mitochondrial function, and regulatory mechanisms, including non-coding variants.
- Molecular diagnosis increasingly informs diazoxide responsiveness, the likelihood of focal versus diffuse disease, functional imaging, and surgical decision-making, although genotype must be interpreted alongside clinical and imaging data.
- Investigational therapies (GLP-1 receptor antagonism, long-acting glucagon analogues, insulin-receptor modulation) remain unproven, and gene-based therapies are so far preclinical or proposed.

**What are the implications of the main findings?**

- Integrating genetic testing with clinical phenotyping can enable a more precise, individualized approach to diagnosis, treatment, genetic counselling, and long-term surveillance in congenital hyperinsulinism.
- Continued genomic discovery and functional validation are needed to resolve genetically unexplained cases and support the development of mechanism-based therapies.

**Abstract:**

Background/Objectives: Congenital hyperinsulinism (CHI) is the leading cause of persistent hypoglycaemia in infancy and childhood characterized by dysregulated insulin secretion despite low plasma glucose concentrations. Early recognition and treatment are essential to reduce the risk of neurological injury. Advances in molecular genetics have revealed substantial genetic and phenotypic heterogeneity beyond classical K_ATP_-channel disorders. This review examines the expanding genetic landscape of CHI and the translation of molecular discoveries into precision clinical care. Methods: This narrative review, based on targeted literature searches of PubMed/MEDLINE, Scopus and Embase and expert selection of key publications, synthesizes current evidence on the genetic mechanisms, genotype–phenotype relationships, molecular diagnosis, and clinical management of CHI, with emphasis on established and emerging genetic subtypes and their implications for therapeutic decision-making. Results: More than 30 disease-associated genes have been identified, involving ion channels, metabolic enzymes, transcription factors, membrane transport proteins, mitochondrial pathways, and developmental mechanisms. Major genetic forms involving *ABCC8*, *KCNJ11*, *GLUD1*, *GCK*, *HK1*, *HADH*, *SLC16A1*, *HNF4A*, *HNF1A*, and *FOXA2* illustrate diverse mechanisms of dysregulated β-cell insulin secretion. Recognition of non-coding regulatory variants has further expanded the molecular spectrum and provided new insights into tissue-specific gene regulation. Molecular diagnosis increasingly informs prediction of diazoxide responsiveness, selection for ^18^F-DOPA PET/CT, identification of patients at high risk of focal disease, surgical planning, genetic counselling, and long-term surveillance. Conclusions: Precision medicine is increasingly transforming CHI from a predominantly phenotype-based disorder into a genetically informed clinical entity. Integration of genomic testing with clinical phenotyping and functional assessment can improve diagnostic classification and individualize management. Continued genomic discovery, functional validation, and international collaboration will be important for patients who remain without a molecular diagnosis and for developing mechanism-based therapies.

## 1. Introduction

Congenital hyperinsulinism (CHI) is the most frequent cause of persistent hypoglycaemia in neonates and infants and represents one of the most challenging endocrine emergencies encountered in paediatric practice. The disorder is characterized by dysregulated insulin secretion from pancreatic β-cells despite hypoglycaemia, resulting in persistent suppression of lipolysis and ketogenesis and depriving the developing brain of its principal alternative energy substrates. Consequently, infants with CHI are particularly vulnerable to neuroglycopenic injury, with delayed diagnosis or inadequate treatment predisposing to seizures, developmental delay, visual impairment, epilepsy, and permanent neurocognitive disability. Despite significant advances in neonatal intensive care and glucose monitoring, neurological morbidity remains an important cause of long-term disability among affected children, emphasizing the need for prompt recognition and timely institution of effective therapy [1,2,3,4,5].

Although considered a rare disease, the reported incidence of CHI varies considerably across different populations, reflecting differences in founder mutations, consanguinity, and access to specialized diagnostic services. Population-based studies estimate an incidence of approximately 1 in 27,000–50,000 live births, whereas substantially higher frequencies have been reported in genetically isolated populations and regions with high rates of consanguineous marriages [6,7]. Clinically, CHI exhibits remarkable heterogeneity. While many infants present within the first few hours or days of life with severe hypoglycaemia requiring high glucose infusion rates, others manifest later in infancy or childhood with recurrent fasting hypoglycaemia, protein-induced hypoglycaemia, or exercise-associated symptoms. Disease severity likewise spans a broad spectrum, ranging from transient forms that resolve spontaneously to diffuse or focal pancreatic disease requiring prolonged medical therapy or surgical intervention. Such diversity cannot be adequately explained by biochemical evaluation alone and reflects the complex molecular architecture underlying insulin secretion.

The understanding of CHI has undergone a remarkable transformation over the past three decades. Historically regarded as a single clinicopathological entity characterized by inappropriate insulin secretion, CHI is now recognized as a genetically heterogeneous group of disorders arising from defects in multiple pathways regulating β-cell stimulus–secretion coupling. These include abnormalities of ATP-sensitive potassium (K_ATP_) channels, enzymes involved in amino acid, glucose and fatty acid metabolism, monocarboxylate transport, transcriptional regulators of pancreatic development and β-cell differentiation, mitochondrial function, and several syndromic developmental pathways. To date, more than thirty genes have been implicated, each contributing distinct molecular mechanisms and clinical phenotypes [8,9,10,11]. This expanding genetic landscape has not only enhanced understanding of β-cell physiology but has also revealed the remarkable biological diversity underlying disorders previously grouped under a single clinical diagnosis.

Perhaps the greatest impact of these discoveries has been the transition of molecular genetics from a purely diagnostic tool to a central component of precision medicine in CHI. Identification of the underlying genetic defect now informs almost every major clinical decision encountered during patient management. Beyond confirming the diagnosis, genetic testing can predict the likelihood of diazoxide responsiveness, distinguish diffuse from focal pancreatic disease, identify patients who are likely to benefit from functional imaging with ^18^F-DOPA PET/CT, guide the extent of surgical resection, estimate recurrence risks for affected families, and facilitate surveillance for syndromic manifestations or later metabolic complications such as diabetes mellitus [12,13,14]. Consequently, molecular diagnosis has evolved from being a confirmatory investigation performed late in the diagnostic pathway to an early investigation that directly influences therapeutic strategy and clinical outcomes.

The rapid pace of gene discovery has simultaneously expanded and complicated the clinical spectrum of CHI. While pathogenic variants in *ABCC8* and *KCNJ11* remain the predominant causes worldwide, advances in next-generation sequencing have uncovered an increasing number of rare and novel disease-causing genes affecting diverse aspects of β-cell biology. Furthermore, considerable phenotypic variability, incomplete penetrance, mosaicism, epigenetic mechanisms, and the continued identification of deep intronic and regulatory variants illustrate that the relationship between genotype and phenotype is often more complex than initially appreciated [15]. Parallel advances in functional genomics, molecular imaging, and transcriptomic technologies are further refining disease classification and offering new opportunities for individualized therapeutic approaches.

These developments have fundamentally altered the approach to children with CHI. Rather than viewing genetic abnormalities simply as etiological explanations, clinicians increasingly use molecular information to stratify disease, individualize therapy, anticipate complications, and counsel families regarding prognosis and recurrence risk. As precision medicine continues to evolve, integrating molecular genetics with clinical, biochemical, radiological, and pathological findings has become essential for optimizing patient care.

In this review, we examine the evolving genetic landscape of congenital hyperinsulinism from a clinically relevant perspective. Rather than presenting a catalogue of disease-causing genes, we discuss how advances in molecular genetics have reshaped current understanding of β-cell biology, influenced diagnostic algorithms, informed therapeutic decision-making, and paved the way toward precision medicine. We also highlight emerging genetic mechanisms, syndromic forms of CHI, current challenges in genotype–phenotype interpretation, and future directions that are likely to further transform the care of children with this complex disorder.

This is a narrative review based on targeted literature searches and expert selection of key publications. PubMed/MEDLINE, Scopus and Embase were searched from database inception to 2026 for articles published in English. The principal search terms, combined using Boolean operators were “congenital hyperinsulinism”, “hyperinsulinaemic hypoglycaemia”, and “persistent hyperinsulinaemic hypoglycaemia of infancy”, together with “genetics”, “*ABCC8*”, “*KCNJ11*”, “genotype-phenotype”, “diazoxide”, “18F-DOPA PET/CT”, “next-generation sequencing”, and “precision medicine”. Reference lists of key articles and guidelines were also screened manually. Large cohort studies, consensus guidelines, and landmark functional studies were prioritized; single case reports were included only for rare genes and syndromes for which larger series were unavailable. Evidence was graded narratively into three tiers. Established findings are supported by consensus guidelines, replicated cohort data, or controlled trials; preliminary findings rest on small, single-arm, or early-phase studies, case series, or unreplicated reports; and proposed strategies are conceptual or preclinical, without clinical data, or represent the author’s own suggestions. The labels used in the tables map onto these tiers as follows: “Established” corresponds to established; “Limited” and “investigational” to preliminary; and “Preclinical/proposed” to proposed. “Disputed” denotes genes for which causality has not been consistently replicated. The review was prepared with reference to the SANRA quality criteria for narrative reviews [16]. Titles and abstract were screened by three authors and disagreements were resolved by consensus/the senior authors. No formal risk-of-bias tool was applied and selection bias cannot be excluded.

## 2. Genetics as the Foundation of Precision Medicine in CHI

The management of congenital hyperinsulinism has undergone a paradigm shift over the past two decades, driven largely by advances in molecular genetics. Historically, the diagnosis and management of CHI relied primarily on clinical presentation, biochemical evaluation, and response to medical therapy. Although these remain fundamental components of patient assessment, they often fail to predict disease severity, treatment responsiveness, or the underlying pancreatic pathology. The incorporation of genetic testing into routine clinical practice has transformed this approach, enabling clinicians to move beyond phenotypic diagnosis toward individualized patient management based on the underlying molecular defect [17,18].

Molecular diagnosis now extends beyond establishing the cause of disease and provides clinically actionable information at several stages of care. It can help distinguish transient from persistent hyperinsulinism, inform the likelihood of diazoxide responsiveness, and identify patients at high risk of focal disease who need functional imaging such as ^18^F-DOPA PET/CT and support genetic counselling, recurrence risk estimation, and recognition of syndromic forms requiring multidisciplinary follow-up [19,20,21,22].

One of the most significant contributions of molecular genetics has been its ability to explain the marked clinical heterogeneity observed in CHI. Children with seemingly similar biochemical profiles may exhibit vastly different clinical courses depending on the affected molecular pathway. For example, pathogenic variants involving the K_ATP_ channel are frequently associated with severe neonatal hypoglycaemia and diazoxide-unresponsive disease, whereas defects affecting metabolic enzymes or transcriptional regulators often demonstrate distinct biochemical signatures, variable age at presentation, differing responses to medical therapy, and unique long-term outcomes. Thus, genotype frequently provides prognostic information that cannot be reliably inferred from clinical presentation alone [10,11,18].

Genetic testing has also changed surgical decision-making. Distinguishing focal from diffuse disease is a key determinant of management, because focal lesions may be cured by limited resection whereas diffuse disease often requires near-total pancreatectomy with its attendant risks. A paternally inherited recessive K_ATP_ variant raises the probability of focal disease but does not establish it, and must be interpreted alongside functional imaging and clinical findings [23].

Beyond immediate therapeutic implications, molecular diagnosis increasingly informs long-term patient management. Certain genetic subtypes are associated with later development of diabetes mellitus, persistent metabolic abnormalities, exercise- or protein-induced hypoglycaemia, or extra-pancreatic manifestations involving the liver, heart, kidneys, or central nervous system. Early recognition of these genotype-specific associations permits anticipatory surveillance and individualized follow-up strategies extending well beyond infancy. Furthermore, the identification of pathogenic variants enables cascade screening of family members and facilitates informed reproductive counselling, particularly in populations with increased rates of consanguinity or founder mutations [24,25].

Despite these advances, important challenges remain. Approximately one-third to one-half of patients with clinically confirmed CHI still lack an identifiable molecular diagnosis despite comprehensive genetic testing, suggesting that additional disease-causing genes, non-coding variants, epigenetic mechanisms, and complex regulatory pathways remain to be discovered. Moreover, variable penetrance, oligogenic inheritance, mosaicism, and variants of uncertain significance continue to complicate genotype–phenotype interpretation in routine clinical practice. As genomic technologies evolve, combining molecular findings with functional studies and detailed clinical phenotyping will be essential for realizing the full potential of precision medicine in CHI [15].

Taken together, these developments underscore that genetics is no longer simply one component of the diagnostic evaluation of CHI but rather the framework upon which contemporary disease classification, therapeutic decision-making, prognostication, and family counselling are increasingly based. Understanding how different molecular pathways disrupt β-cell physiology is therefore fundamental to appreciating the diverse clinical manifestations of CHI and forms the basis for the discussion in the following sections.

## 3. Molecular Basis of β-Cell Dysfunction in CHI

### 3.1. Normal Regulation of Insulin Secretion: A Framework for Understanding CHI

Insulin secretion by pancreatic β-cells is governed by a tightly coordinated network of nutrient sensing, intracellular metabolism, ion channel activity, and stimulus–secretion coupling that ensures insulin is released only when circulating glucose concentrations are sufficient to meet the metabolic demands of peripheral tissues. This exquisitely regulated process enables β-cells to continuously integrate nutritional, hormonal, and neuronal signals while maintaining glucose homeostasis. Congenital hyperinsulinism arises when genetic defects disrupt one or more components of this regulatory network, resulting in inappropriate insulin secretion despite hypoglycaemia.

Under physiological conditions, glucose enters the β-cell predominantly through glucose transporters and undergoes phosphorylation by glucokinase, the principal glucose sensor of the pancreatic β-cell. Subsequent glycolysis and mitochondrial oxidative phosphorylation increase intracellular ATP production, leading to closure of K_ATP_ channels located on the β-cell membrane. Membrane depolarization that follows triggers opening of voltage-gated calcium channels, allowing calcium influx and initiating exocytosis of insulin-containing secretory granules. This glucose-stimulated insulin secretion pathway represents the central mechanism maintaining normal glucose homeostasis and provides the molecular basis for understanding many forms of CHI [26,27,28].

Although glucose remains the dominant physiological stimulus, insulin secretion is influenced by numerous additional metabolic pathways. Amino acid metabolism, fatty acid β-oxidation, mitochondrial function, and monocarboxylate transport all contribute to intracellular ATP generation or metabolic signaling, thereby modulating β-cell excitability. Equally important are transcription factors responsible for pancreatic development and maintenance of β-cell identity, as well as proteins involved in mitochondrial integrity, cellular differentiation, and intracellular signaling. Consequently, defects affecting seemingly unrelated cellular processes may ultimately converge on a common phenotype of dysregulated insulin secretion.

The remarkable genetic heterogeneity observed in CHI reflects disruption of these diverse physiological pathways rather than a single molecular defect. While pathogenic variants involving K_ATP_ channel genes account for the largest proportion of genetically confirmed cases, an increasing number of disease-causing variants have been identified in genes regulating nutrient sensing, intermediary metabolism, transcriptional control, membrane transport, and cellular development. These discoveries have expanded the understanding of CHI beyond a disorder of ion-channel dysfunction to one encompassing multiple interconnected mechanism governing β-cell physiology [10,11,15,17,18].

For clinical practice, classifying CHI according to the affected biological pathway offers advantages over considering individual genes in isolation. Disorders affecting the same molecular pathway frequently exhibit shared biochemical characteristics, therapeutic responses, and clinical behavior despite involving different genes. Conversely, variants affecting distinct pathways may require fundamentally different diagnostic approaches and management strategies. Organizing CHI according to molecular mechanisms therefore provides a more clinically meaningful framework for understanding genotype–phenotype relationships and precision medicine than a simple catalogue of disease-causing genes [29].

Based on current understanding, the genetic causes of CHI can be broadly grouped into five mechanistic categories:

- Disorders of K_ATP_ channel function (*ABCC8*, *KCNJ11*; Section 3.2);
- Abnormalities of nutrient-sensing and intermediary metabolism (*GLUD1*, *GCK*, *HK1*; Section 3.3);
- Abnormalities involving membrane transport and mitochondrial function (*HADH*, *SLC16A1*; Section 3.4 and Section 3.5);
- Defects of transcriptional regulation and pancreatic development (*HNF4α*, *HNF1α*; Section 3.6);
- Syndromic disorders with a broader developmental phenotype (Section 3.7).

The following sections discuss each of these categories, emphasizing the molecular mechanisms through which they disrupt β-cell physiology and their implications for diagnosis, treatment, and long-term clinical management.

### 3.2. Disorders of K_ATP_ Channel Function: The Prototypical Form of CHI

Disorders affecting the K_ATP_ channel constitute the commonest and clinically most significant genetic cause of congenital hyperinsulinism, accounting for nearly half of all genetically confirmed cases and the majority of severe diazoxide-unresponsive disease [10,11,17,18]. Beyond their frequency, K_ATP_ channel defects occupy a central position in the pathophysiology of CHI because they disrupt the final common pathway linking intracellular metabolism to insulin secretion. Consequently, these disorders exemplify how molecular diagnosis directly influences clinical management, including prediction of treatment response, localization of focal pancreatic lesions, and selection of patients for surgical intervention.

The pancreatic β-cell K_ATP_ channel is a hetero-octameric complex composed of four inwardly rectifying potassium channel (Kir6.2) subunits encoded by *KCNJ11* and four regulatory sulfonylurea receptor-1 (SUR1) subunits encoded by *ABCC8*. Under physiological conditions, fluctuations in the intracellular ATP/ADP ratio regulate channel activity, coupling cellular metabolism to membrane excitability. Rising ATP concentrations following glucose metabolism induce channel closure, membrane depolarization, calcium influx through voltage-gated calcium channels, and insulin exocytosis. Conversely, reopening of the K_ATP_ channel during hypoglycaemia restores membrane hyperpolarization and suppresses insulin secretion. Pathogenic variants that impair channel activity abolish this glucose-dependent regulation, resulting in persistent membrane depolarization and inappropriate insulin release irrespective of circulating glucose concentrations [3,26,27,28,30,31,32].

Although *ABCC8* and *KCNJ11* mutations disrupt the same molecular complex, they demonstrate considerable genetic and phenotypic diversity. Pathogenic variants may be inherited in either autosomal recessive or autosomal dominant patterns, while de novo mutations and mosaicism have also been described. Recessive loss-of-function variants typically produce severe neonatal disease characterized by profound hypoglycaemia, markedly elevated glucose requirements, and poor responsiveness to diazoxide. In contrast, dominant variants often retain partial channel activity and may present with milder or later-onset disease, although substantial overlap in clinical severity exists. Functional consequences of individual variants, rather than inheritance pattern alone, frequently determine disease expression, highlighting the complexity of genotype–phenotype relationships [10,11,17,28,33].

Perhaps the greatest clinical significance of K_ATP_ channel genetics lies in its ability to identify patients at high risk of focal forms of CHI. Focal lesions arise through a unique two-hit mechanism involving inheritance of a paternal recessive *ABCC8* or *KCNJ11* variant followed by somatic loss of the maternal allele within a localized region of chromosome 11p15 in pancreatic β-cells. This results in clonal expansion of hyperfunctional β-cells confined to a discrete pancreatic focus while the remaining pancreas remains functionally normal. Recognition of this mechanism has fundamentally altered the diagnostic pathway for CHI, allowing molecular testing to identify children who should undergo lesion localization using ^18^F-DOPA PET/CT or, where available, GLP-1 receptor imaging before surgical exploration [19,20,21,22]. A paternally inherited variant strongly raises, but does not confirm, the likelihood of a focal lesion; localization and exclusion of diffuse disease depend on functional imaging and clinical assessment.

The therapeutic implications of identifying K_ATP_ channel defects are equally profound. Diazoxide, the first-line medical therapy for CHI, acts by binding to the SUR1 subunit and maintaining the K_ATP_ channel in its open state. Consequently, patients with complete loss-of-function variants that abolish channel activity are typically diazoxide unresponsive, whereas those with residual channel function may demonstrate partial or complete responsiveness. Establishing the underlying genotype therefore provides an early indication of the likelihood of successful medical therapy and assists clinicians in avoiding prolonged ineffective treatment while expediting referral for advanced imaging or surgical assessment when appropriate [18,23,33].

Long-term outcomes among patients with K_ATP_ channel defects remain highly variable and depend upon both the underlying molecular defect and the therapeutic strategy employed. Children successfully treated for focal disease following limited pancreatic resection often achieve complete cure without recurrence, whereas those with diffuse disease requiring near-total pancreatectomy remain at substantial risk of persistent hypoglycaemia, insulin-dependent diabetes mellitus, pancreatic exocrine insufficiency, and neurodevelopmental impairment. Even medically managed patients require continued surveillance because the relationship between genotype, β-cell function, and long-term metabolic health continues to evolve with increasing clinical experience [13,14,23,24,25,34].

Collectively, disorders of the K_ATP_ channel illustrate the transition of CHI management from empirical therapy to precision medicine. Few inherited endocrine disorders demonstrate such a direct relationship between molecular diagnosis and clinical decision-making, where identification of a pathogenic variant can simultaneously inform disease mechanism, the likelihood of response to pharmacological therapy, the need for functional imaging, surgical planning, recurrence risk, and long-term prognosis, although each of these would require integration with clinical, biochemical and imaging data. For this reason, K_ATP_ channel disorders remain the benchmark against which emerging genetic causes of CHI continue to be understood.

### 3.3. Disorders of Nutrient Sensing: When β-Cells Misinterpret Metabolic Signals

While K_ATP_ channel defects impair the final common pathway coupling cellular metabolism to membrane excitability, a second major group of CHI arises from abnormalities in intracellular nutrient sensing. These disorders illustrate that inappropriate insulin secretion may occur despite structurally and functionally intact K_ATP_ channels. Instead, pathogenic variants alter metabolic pathways responsible for generating intracellular signals that regulate insulin release, effectively lowering the threshold at which β-cells perceive nutrient availability. Consequently, insulin secretion becomes dissociated from physiological glucose concentrations, resulting in persistent or stimulus-specific hypoglycaemia. Unlike K_ATP_ channel disorders, many nutrient-sensing defects remain responsive to diazoxide because the K_ATP_ channel itself is preserved and capable of pharmacological activation [10,18,26,27,35,36,37].

Among these disorders *GLUD1*, *GCK*, and *HK1* represent distinct but complementary mechanisms through which altered intracellular metabolism promotes inappropriate insulin secretion. Collectively, these conditions have significantly expanded the understanding of β-cell physiology by demonstrating that abnormalities in amino acid metabolism, glucose sensing, or ectopic enzyme expression can independently trigger hyperinsulinism without primary defects in ion-channel function.

#### 3.3.1. *GLUD1*: Dysregulated Amino Acid Metabolism

Activating variants in *GLUD1*, encoding mitochondrial glutamate dehydrogenase (GDH), constitute the second most common monogenic cause of CHI after K_ATP_ channel disorders. GDH catalyzes the oxidative deamination of glutamate to α-ketoglutarate, linking amino acid metabolism to the tricarboxylic acid cycle and ATP generation. Under normal physiological conditions, GDH activity is tightly inhibited by guanosine triphosphate (GTP), preventing excessive insulin secretion following protein ingestion. Pathogenic gain-of-function variants reduce this inhibitory regulation, resulting in constitutively increased enzyme activity, enhanced ATP production, and inappropriate insulin secretion despite low plasma glucose concentrations [10,35,36,37].

The resulting clinical phenotype, commonly referred to as the hyperinsulinism–hyperammonaemia (HI/HA) syndrome, is characterized by fasting hypoglycaemia, exaggerated insulin secretion following protein-rich meals, and persistent asymptomatic hyperammonaemia. Unlike K_ATP_ channel defects, neurological manifestations in HI/HA syndrome are not solely attributable to hypoglycaemia, and epilepsy or developmental abnormalities may occur even in individuals with relatively well-controlled glucose concentrations, suggesting additional effects of altered glutamate metabolism within the central nervous system [10,36,37].

From a therapeutic perspective, most patients with *GLUD1*-related CHI demonstrate an excellent response to diazoxide because K_ATP_ channel function remains intact. Dietary protein modification may further reduce hypoglycaemic episodes, while pancreatectomy is rarely required. Recognition of the characteristic association between hypoglycaemia and persistent hyperammonaemia therefore provides an important biochemical clue that can guide targeted molecular testing and facilitate early diagnosis [10,18,36].

#### 3.3.2. *GCK*: Altered Glucose Sensing

Glucokinase serves as the principal glucose sensor of the pancreatic β-cell, catalyzing the phosphorylation of glucose to glucose-6-phosphate and thereby determining the threshold for glucose-stimulated insulin secretion. Activating variants in *GCK* lower this glucose threshold, causing insulin secretion to commence at plasma glucose concentrations that would normally suppress β-cell activity. Consequently, affected individuals develop hyperinsulinaemic hypoglycaemia despite otherwise preserved insulin secretory pathways [26,38,39].

The clinical spectrum associated with activating *GCK* variants is remarkably broad. Some individuals present during the neonatal period with severe persistent hypoglycaemia requiring pharmacological treatment, whereas others experience only mild fasting hypoglycaemia detected incidentally later in life. Considerable intrafamilial variability has also been reported, indicating that additional genetic or environmental modifiers influence disease expression. Unlike many other forms of CHI, genotype–phenotype correlations remain relatively weak, limiting prediction of disease severity based solely on the underlying variant [38,39].

As K_ATP_ channel function remains preserved, many patients demonstrate partial or complete responsiveness to diazoxide, although treatment requirements vary considerably according to disease severity. Recognition of activating *GCK* variants is clinically important because these patients rarely require surgical intervention and generally have favorable long-term outcomes with appropriate medical management.

#### 3.3.3. *HK1*: Expanding the Spectrum of β-Cell Glucose Dysregulation

The recent identification of pathogenic regulatory variants involving *HK1* has provided important insights into previously unrecognized mechanisms of CHI. Unlike glucokinase, *HK1* is normally silenced in mature pancreatic β-cells, thereby ensuring that glucokinase remains the dominant physiological glucose sensor. Disease-causing non-coding variants disrupt this developmental silencing, leading to inappropriate expression of hexokinase 1 within β-cells. Because HK1 possesses a substantially higher affinity for glucose than glucokinase, insulin secretion is initiated at markedly lower glucose concentrations, producing persistent hyperinsulinaemic hypoglycaemia [40,41].

The discovery of *HK1* represents a major advance in the genetics of CHI for two reasons. First, it highlights the importance of non-coding regulatory variants in disease pathogenesis, emphasizing that conventional exon-focused sequencing strategies may fail to identify clinically significant mutations. Second, it demonstrates that disruption of normal developmental gene repression, rather than alteration of protein structure itself, may be sufficient to produce severe hyperinsulinism. These observations have broadened current concepts of β-cell biology and underscore the increasing role of whole-genome sequencing and functional genomic approaches in patients lacking a molecular diagnosis [40,41].

Taken together, disorders of nutrient sensing illustrate that CHI encompasses far more than abnormalities of ion-channel function. By affecting distinct metabolic checkpoints regulating amino acid metabolism, glucose sensing, and developmental control of enzyme expression, *GLUD1*, *GCK*, and *HK1* demonstrate how diverse intracellular pathways converge upon a common phenotype of dysregulated insulin secretion. Clinically, these disorders share a greater likelihood of diazoxide responsiveness than K_ATP_ channel disease while exhibiting characteristic biochemical signatures that can facilitate targeted diagnostic evaluation and individualized management.

### 3.4. Fatty Acid Oxidation and Mitochondrial Metabolism: The Role of HADH

The contribution of mitochondrial fatty acid oxidation to insulin secretion was initially underappreciated until the identification of pathogenic variants in *HADH* (also known as SCHAD) encoding short-chain 3-hydroxyacyl-CoA dehydrogenase. Although uncommon, *HADH*-associated CHI provided critical evidence that β-cell metabolism extends beyond glucose sensing and K_ATP_ channel regulation. Instead, mitochondrial fatty acid oxidation directly influences nutrient-induced insulin secretion through interactions with amino acid metabolism and glutamate dehydrogenase (GDH), thereby broadening current understanding of β-cell stimulus–secretion coupling [10,42,43,44].

HADH is a mitochondrial enzyme involved in the β-oxidation of fatty acids, catalysing the conversion of short-chain 3-hydroxyacyl-CoA intermediates. Early descriptions of affected children suggested that defective fatty acid oxidation alone was responsible for hyperinsulinism. However, subsequent experimental studies demonstrated that the pathophysiological mechanism is considerably more complex. In pancreatic β-cells, SCHAD (encoded by *HADH*) interacts directly with glutamate dehydrogenase, functioning as a negative regulator of GDH activity independent of its enzymatic role in fatty acid oxidation. Loss of *HADH* therefore results in inappropriate activation of GDH, enhanced oxidative metabolism, increased ATP generation, and excessive insulin secretion despite hypoglycaemia [10,42,43,44].

Clinically, *HADH*-related CHI shares several characteristics with *GLUD1*-associated disease but also exhibits important distinguishing features. Affected individuals frequently present during infancy with recurrent fasting hypoglycaemia and may demonstrate marked sensitivity to protein-rich meals, reflecting enhanced leucine-stimulated insulin secretion. Unlike *GLUD1*-associated hyperinsulinism, however, persistent hyperammonaemia is absent because GDH itself remains structurally normal. Recognition of this biochemical distinction provides an important diagnostic clue when evaluating protein-sensitive forms of congenital hyperinsulinism [10,42,43,44].

The majority of reported patients exhibit autosomal recessive inheritance, with pathogenic variants resulting in complete or near-complete loss of *HADH* function. Although the disorder is rare globally, a higher prevalence has been reported in populations with increased rates of consanguinity, highlighting the importance of considering *HADH* deficiency in children from these settings who present with diazoxide-responsive hyperinsulinaemic hypoglycaemia. Molecular confirmation not only establishes the diagnosis but also facilitates family screening and genetic counselling [10,42,43,44].

From a therapeutic perspective, preservation of K_ATP_ channel function explains the generally favorable response of *HADH*-associated CHI to diazoxide. Most patients achieve satisfactory glycaemic control with medical therapy combined with avoidance of prolonged fasting and, in selected cases, dietary modification to reduce protein-induced hypoglycaemia. Pancreatectomy is rarely required, and long-term prognosis is generally favorable when hypoglycaemia is recognized early and treated appropriately. Nevertheless, delayed diagnosis may still result in preventable neurological injury, underscoring the importance of early recognition despite the rarity of this disorder [10,17,42,43,44].

The discovery of *HADH* deficiency significantly expanded the conceptual framework of congenital hyperinsulinism by demonstrating that defects in mitochondrial metabolism can dysregulate insulin secretion through mechanisms distinct from primary ion-channel abnormalities. Furthermore, elucidation of the functional interaction between *HADH* and GDH has reinforced the concept that β-cell nutrient sensing depends upon coordinated metabolic networks rather than isolated enzymatic pathways. These insights continue to influence the interpretation of newly identified metabolic forms of CHI and illustrate the close relationship between intermediary metabolism and endocrine regulation.

### 3.5. Disorders of Membrane Transport: Exercise-Induced Hyperinsulinism Due to SLC16A1

The identification of pathogenic variants in *SLC16A1*, encoding the monocarboxylate transporter-1 (MCT1), introduced a novel mechanism of congenital hyperinsulinism in which disease results not from structural defects in metabolic enzymes or ion channels but from inappropriate expression of an otherwise physiologically normal transporter within pancreatic β-cells. This discovery demonstrated that maintenance of β-cell identity depends not only on the expression of specific genes but also on the selective repression of genes that could otherwise disrupt glucose-dependent insulin secretion [45,46,47].

Under normal physiological conditions, MCT1 facilitates the transport of monocarboxylates such as lactate and pyruvate across cell membranes and is widely expressed in metabolically active tissues. Mature pancreatic β-cells, however, normally suppress *SLC16A1* expression, thereby preventing circulating lactate and pyruvate from serving as alternative metabolic substrates for insulin secretion. This β-cell-specific silencing ensures that insulin release remains primarily dependent on circulating glucose concentrations rather than fluctuations in other metabolites generated during exercise or anaerobic metabolism [45,46,47].

Pathogenic regulatory variants affecting *SLC16A1* disrupt this physiological silencing, leading to ectopic expression of MCT1 in pancreatic β-cells. Consequently, lactate and pyruvate generated during vigorous anaerobic exercise are transported into β-cells, where they undergo mitochondrial metabolism and stimulate ATP production independent of blood glucose concentration. The resulting closure of K_ATP_ channels initiates membrane depolarization and insulin secretion despite normal or declining plasma glucose levels, producing the characteristic phenotype of exercise-induced hyperinsulinaemic hypoglycaemia [45,46,47].

Clinically, affected individuals typically present later than patients with classical neonatal forms of congenital hyperinsulinism. Episodes of hypoglycaemia are characteristically precipitated by short periods of intense anaerobic exercise rather than prolonged fasting, and symptoms often become more apparent during late childhood or adolescence when participation in competitive sports increases. Recognition of this distinctive clinical pattern is essential, as routine fasting studies may be normal and diagnosis often depends upon careful correlation of symptoms with physical activity [45,46,47].

Unlike disorders caused by structural defects in K_ATP_ channels, *SLC16A1*-related hyperinsulinism generally responds well to diazoxide because K_ATP_ channel function remains intact. Management additionally focuses on lifestyle modification, including avoidance of strenuous anaerobic exercise when feasible, optimization of pre-exercise carbohydrate intake, and individualized counselling regarding physical activity. Most patients can achieve satisfactory long-term glycaemic control without surgical intervention, emphasizing the value of early molecular diagnosis in preventing unnecessary investigations and inappropriate treatment [18,45,46,47].

Beyond its clinical relevance, the discovery of *SLC16A1*-associated hyperinsulinism has had important implications for β-cell biology. It established that congenital hyperinsulinism may arise through failure of tissue-specific gene repression rather than alteration of protein function itself, thereby expanding the spectrum of pathogenic mechanisms beyond conventional coding mutations. This concept has subsequently gained increasing importance with the identification of additional regulatory variants in disorders such as *HK1*-associated hyperinsulinism, highlighting the growing contribution of epigenetic and transcriptional regulation to the molecular pathogenesis of CHI [40,41,45,46,47].

### 3.6. Transcription Factor Defects: Developmental Regulation of β-Cell Function

The identification of pathogenic variants in transcription factors associated with CHI include abnormalities in pancreatic development, β-cell differentiation, and gene regulation. Unlike defects affecting ion channels or metabolic enzymes, transcription factor variants disrupt complex genetic networks that govern β-cell maturation, nutrient sensing, and insulin secretory function resulting in variable and evolving phenotypes [48,49].

Among the transcription factor-associated forms of CHI, *HNF4α* and *HNF1α* are the best characterized. These genes encode hepatocyte nuclear factors that regulate the expression of numerous genes involved in glucose transport, mitochondrial metabolism, insulin biosynthesis, and stimulus–secretion coupling [48,49,50]. Consequently, pathogenic variants impair coordinated regulation of multiple downstream pathways rather than disrupting a single metabolic step.

An intriguing feature of *HNF4α* and *HNF1α*-associated hyperinsulinism is the changing clinical phenotype across the lifespan. Many affected infants present with transient or persistent neonatal hyperinsulinaemic hypoglycaemia, frequently accompanied by increased birth weight due to fetal hyperinsulinism. However, as β-cell function progressively declines with age, the same pathogenic variants may later result in maturity-onset diabetes of the young (MODY), illustrating the remarkable duality of these transcription factor disorders [50,51,52,53].

Identification of an *HNF4α* or *HNF1α* pathogenic variant not only explains the presenting episode of hypoglycaemia but also permits anticipatory surveillance for diabetes mellitus in adolescence or adulthood. Furthermore, the autosomal dominant inheritance pattern frequently observed in these disorders emphasizes the importance of obtaining a detailed multigenerational family history when evaluating children with congenital hyperinsulinism [18,50,51,52,53].

Pathogenic variants in *FOXA2*, a master regulator of foregut and pancreatic development, have been associated with hyperinsulinism accompanied by structural abnormalities of the pituitary gland, craniofacial anomalies, and variable endocrine deficiencies. These observations reinforce the concept that congenital hyperinsulinism may represent one manifestation of broader developmental disorders affecting multiple organ systems [54,55].

Most transcription factor-associated forms of CHI retain functional K_ATP_ channels and therefore demonstrate variable but generally favorable responses to diazoxide. Nevertheless, therapeutic requirements are highly heterogeneous, reflecting differences in the underlying molecular defect and the extent of β-cell dysfunction. Long-term follow-up remains essential, not only to monitor glycaemic control but also to detect evolving endocrine manifestations and the potential transition from hyperinsulinism to diabetes mellitus.

The major monogenic forms of CHI are summarized in Table 1, highlighting the underlying molecular defects, inheritance patterns, characteristic clinical features, expected response to diazoxide, and the strength of evidence supporting each gene. *UCP2* and *INSR* are included for completeness but should be interpreted with caution. Dominant *UCP2* variants were initially reported in diazoxide-responsive CHI, but analysis of large sequencing cohorts has not consistently supported a causal role [56]. *INSR* variants have been described in patients whose hyperinsulinaemic hypoglycaemia later evolves into insulin resistance, but the mechanism remains uncertain and few cases have been reported.

### 3.7. Emerging Genetic Mechanisms and Syndromic Forms of CHI

The rapid expansion of genomic sequencing technologies has substantially broadened the molecular landscape of CHI. An increasing number of rare disease-causing genes affecting calcium signaling, mitochondrial homeostasis, protein glycosylation, intracellular trafficking and developmental pathways have highlighted the remarkable complexity of β-cell biology [18,57,58,59].

One important group comprises disorders affecting ion transport and membrane excitability beyond the K_ATP_. Activating variants in *CACNA1D*, encoding the α1-subunit of the L-type voltage-gated calcium channel (CaV1.3), result in persistent calcium influx and inappropriate insulin exocytosis independent of physiological metabolic regulation. In contrast to isolated hyperinsulinism, affected individuals frequently demonstrate multisystem developmental and endocrine abnormalities [57,58,59].

Pathogenic variants in *PMM2* are associated with hyperinsulinaemic hypoglycaemia occurring in conjunction with polycystic kidney disease and congenital hepatic fibrosis, whereas defects in *PGM1* may produce a complex phenotype characterized by fasting hypoglycaemia, endocrine abnormalities, cardiomyopathy, hepatopathy, and impaired protein glycosylation. Unlike classical monogenic CHI, these disorders illustrate that dysregulated insulin secretion may arise secondary to generalized metabolic dysfunction rather than isolated β-cell abnormalities. Recognition of these multisystem phenotypes is therefore essential for establishing an accurate diagnosis and coordinating long-term care across multiple specialties [60,61,62,63].

Mitochondrial dysfunction has emerged as another important mechanism underlying congenital hyperinsulinism. Pathogenic variants affecting genes involved in mitochondrial energy production, oxidative phosphorylation, and intermediary metabolism disrupt ATP generation and intracellular signaling pathways that regulate insulin secretion. Although these conditions remain individually rare, their identification has reinforced the concept that intact mitochondrial function is fundamental to β-cell glucose sensing [18,30,60,61,62,63].

Perhaps the most significant recent advance has been the recognition of non-coding and regulatory variants as important contributors to CHI. The discovery of regulatory mutations involving *HK1* and *SLC16A1* demonstrated that disruption of tissue-specific gene silencing may be sufficient to produce severe hyperinsulinism despite preservation of normal protein structure [18,30,41,42,46,47,48].

The expanding genetic landscape of CHI reinforces the need for comprehensive molecular testing and pathway-based classification. Although individually rare, these disorders continue to refine our understanding of β-cell biology and support increasingly personalized approaches to diagnosis and management.

Syndromic forms of CHI are summarized in Table 2, emphasizing their underlying genetic defects, pathogenic mechanisms, associated clinical features, and diagnostic implications. Genetic disorders that cause hypoglycaemia without inappropriate insulin secretion, such as *AKT2*-related disorder, can mimic CHI and are listed separately in Table 3.

## 4. Translating Genetics into Clinical Decision-Making

The integration of molecular diagnostics into routine clinical practice as well as the expanded understanding of genotype–phenotype relationships has transformed genetic testing from a confirmatory investigation into an essential component of clinical decision-making. Identification of the underlying molecular defect increasingly informs therapeutic selection, the likelihood of response to medical therapy, guides imaging strategies, contributes to decisions about surgical intervention and facilitates long-term surveillance for associated endocrine and neurodevelopmental complications [17,18,35]. The approaches described in this section reflect practice supported by international consensus guidelines [18] and cohort data unless otherwise indicated.

Optimal use of genetic testing requires close collaboration among paediatric endocrinologists, clinical geneticists, radiologists, surgeons, and genetic counsellors, so that treatment is tailored to the underlying disease mechanism and not to the clinical phenotype alone.

### 4.1. Genotype as a Predictor of Diazoxide Responsiveness

Diazoxide remains the first-line pharmacological therapy for most children with CHI. Although the response varies considerably according to the underlying molecular defect, genetic testing increasingly allows prediction of treatment response before prolonged therapeutic trials [17,18,35].

As described in Section 3.2, children with recessive inactivating variants in *ABCC8* and *KCNJ11* that abolish K_ATP_ channel function are typically unresponsive to diazoxide, and continuing high-dose treatment may delay definitive management. Patients with dominant variants that retain residual channel activity may respond partially or completely, so genetic findings should be interpreted alongside functional and clinical data and not by inheritance pattern alone.

In contrast, disorders affecting nutrient sensing and intermediary metabolism as well as membrane transport and mitochondrial function, including *GLUD1* and *GCK*, as well as *HADH* and *SLC16A1*, respectively, generally preserve K_ATP_ channel integrity and therefore exhibit a substantially higher likelihood of diazoxide responsiveness. Although the response varies between disorders and individual variants, molecular diagnosis frequently provides clinicians with realistic expectations regarding treatment success and facilitates counselling of families early in the disease course.

Importantly, genotype should complement rather than replace careful clinical assessment. Variability in expressivity, novel variants of uncertain significance, and incomplete genotype–phenotype correlations mean that treatment decisions should always incorporate biochemical findings, glucose requirements, and the patient’s clinical response [17,18].

### 4.2. Selecting Patients for Functional Imaging and Surgery

Pancreatic surgery is reserved for medically unresponsive CHI, particularly when focal disease is suspected. As described in Section 3.2, detection of a paternally inherited recessive *ABCC8* or *KCNJ11* pathogenic variant strongly raises the possibility of focal CHI, prompting localization with ^18^F-DOPA PET/CT before operative intervention [18,19,20,21,22,23,24]. In contrast, biallelic recessive or dominant pathogenic K_ATP_ variants are usually associated with diffuse disease, although exceptions, including mosaic or atypical presentations, occur. Integration of molecular findings with functional imaging has therefore substantially reduced unnecessary extensive pancreatic resections while improving the identification of children who may achieve complete cure following limited lesionectomy [22,23,24,35].

Despite these advances, molecular findings should not be interpreted in isolation. Discordance between genotype, imaging, and clinical phenotype occasionally occurs, particularly in patients with mosaicism, atypical inheritance, or previously undescribed variants. Optimal surgical decision-making therefore requires multidisciplinary evaluation integrating genetic, radiological, biochemical, and histopathological findings [18,35].

### 4.3. Genetic Counselling and Long-Term Follow-Up

The value of molecular diagnosis extends beyond acute management. Identification of the causative variant establishes the mode of inheritance, enables recurrence-risk counselling, facilitates cascade testing of at-risk relatives, and permits informed reproductive planning. This is particularly relevant for autosomal dominant disorders such as *HNF4α*, *HNF1α*, and *GLUD1*, in which apparently unaffected family members may carry pathogenic variants with variable clinical expression [17,18,49,50,51,52,53,54,55].

Equally important is the recognition that several genetic forms of CHI require disease-specific long-term surveillance. Children with *HNF4α* or *HNF1α*-associated hyperinsulinism should be monitored for the later development of maturity-onset diabetes of the young, whereas individuals with syndromic forms may require ongoing assessment for neurological, renal, hepatic, cardiac, or endocrine complications. Genetic diagnosis therefore informs both immediate management and disease specific long-term surveillance [13,14,15,18,49,50,51,52,53,54,55].

## 5. Emerging and Investigational Therapies

Management of CHI still relies on a small number of agents that suppress insulin secretion regardless of the underlying genotype. Diazoxide remains the only approved drug and is first-line; octreotide, lanreotide and continuous glucagon are widely used off-label, and pancreatectomy is reserved for medically unresponsive disease [18,23,121]. Approximately half of patients respond inadequately to available medical therapy [23,121], which has driven the development of the agents below. Their level of evidence differs markedly and is summarized in Table 4.

### 5.1. Investigational Agents in Clinical Development

Avexitide (exendin-(9-39)) antagonizes the GLP-1 receptor. In an open-label crossover study of 16 children with hyperinsulinism, almost all with genetically confirmed K_ATP_ channel disease, short-term intravenous exendin-(9-39) increased fasting glucose in one dosing group, reduced the likelihood of fasting hypoglycaemia by 76–84% in the two higher-dose groups, and reduced protein-induced hypoglycaemia by 82% in the eight children who completed the challenge studies, with no serious adverse events [122]. Postprandial glucose also rose without a measurable fall in insulin secretion, which the authors attributed partly to extra-pancreatic effects. and has been evaluated in children with CHI during fasting and after meal and protein challenges [123]. Evidence in CHI is limited to small, short-term studies of intravenous administration, and multiple-dose subcutaneous regimens have yet to be evaluated. Dasiglucagon, a stable glucagon analogue suited to continuous subcutaneous infusion, has been evaluated in two randomized trials. In infants under 12 months of age dependent on intravenous glucose, it reduced the mean glucose infusion rate by 55% compared with placebo over 48 h [124]. In children aged 3 months to 12 years, the trial did not meet its primary endpoint of self-monitored hypoglycaemia episodes, although post hoc analysis of blinded continuous glucose monitoring showed a 43% reduction in hypoglycaemia; CGM metrics have not been validated in CHI [125]. Ersodetug, a monoclonal antibody that modulates the insulin receptor, acts downstream of the β-cell and is therefore theoretically independent of genotype. In an open-label phase 2b study of 23 patients with CHI on standard therapy, add-on intravenous ersodetug reduced hypoglycaemia events and time in hypoglycaemia by median 59% and 54% from baseline, and was generally well tolerated, although the primary endpoint of time in range did not change significantly. In the randomized, placebo-controlled phase 3 trial (sunRIZE; 55 randomized participants, 24 weeks), the primary and key secondary endpoints were not met. Reductions in hypoglycaemia from baseline were consistent in direction but differed significantly from placebo only at week 16 by continuous glucose monitoring, and the investigators attributed the result partly to a study effect [126]. Four participants discontinued because of adverse events, three of them allergic reactions.; these results have so far been presented only in abstract form. Pasireotide, a somatostatin analogue with higher SSTR5 affinity, has been used in isolated case reports [127], and more selective somatostatin-receptor agonists are under evaluation. Sirolimus has been used in severe diazoxide- and octreotide-unresponsive disease, but serious adverse effects have prompted calls for caution [128].

### 5.2. Preclinical and Proposed Genetic Strategies

There are, to our knowledge, no published clinical reports of gene therapy, gene editing or RNA-based treatment in CHI. Current work is preclinical, including genome-edited stem-cell-derived islet models of K_ATP_-channel disease, and studies of pharmacological chaperones that correct K_ATP_ channel trafficking defects [129]. Such approaches remain conceptual, and challenges include delivery to β-cells, the range of pathogenic mechanisms (loss versus gain of function, recessive versus dominant), and safety in infants. We therefore consider them proposals for future research, not treatment options.

## 6. Future Directions: Advancing Precision Medicine in CHI

This section combines developments that are already established or emerging in practice with the author’s proposals for future clinical strategy; the level of evidence is indicated for each. Despite major advances in the molecular genetics of CHI, important challenges remain before precision medicine can be fully realized in routine clinical practice. A substantial proportion of patients with clinically confirmed CHI continue to lack a definitive molecular diagnosis, highlighting the presence of undiscovered disease mechanisms and emphasizing the limitations of current diagnostic approaches [18,30,63].

### 6.1. Diagnostic Strategies: Established and Emerging

One of the most important developments has been the increasing adoption of next-generation sequencing (NGS) as the first-line genetic investigation for children with persistent hyperinsulinaemic hypoglycaemia. Targeted gene panels currently provide rapid and cost-effective molecular diagnosis for the majority of patients with monogenic disease while simultaneously identifying variants across multiple causative genes. However, panel-based testing remains limited to known disease genes and may miss structural and regulating variants as well as other non-coding alterations. The discovery of pathogenic regulatory variants involving *HK1* and *SLC16A1* has underscored the importance of these previously overlooked mechanisms and strengthened the case for broader genomic approaches in unresolved cases [18,40,41,45]. First line NGS panel testing is established; broader genomic approaches are emerging and are so far used mainly in unresolved cases.

### 6.2. Functional Genomics and Mechanism-Based Therapy: Preliminary and Proposed

Whole-exome sequencing and, increasingly, whole-genome sequencing are beginning to address these diagnostic gaps. In addition to identifying novel coding variants, whole-genome sequencing enables interrogation of non-coding regions, structural variation, copy number changes, and complex genomic rearrangements that are inaccessible using conventional sequencing strategies. The incorporation of transcriptomic, epigenomic, and metabolomic data may further improve variant interpretation and facilitate the discovery of new molecular pathways regulating β-cell function. Beyond diagnosis, advances in functional genomics are reshaping the interpretation of genetic variation. Functional genomic platforms, including stem-cell models and genome-editing technologies are increasingly used to characterize variants of uncertain significance and establish genotype–phenotype relationships. These approaches are expected to improve diagnostic confidence while simultaneously identifying novel therapeutic targets that extend beyond the K_ATP_ [18].

Beyond the investigational agents summarized in Section 5, the authors propose that future therapy should move towards mechanism-based treatment matched to molecular diagnosis, for example by targeting ion-channel trafficking or function, intracellular signalling, or gene regulation. These strategies remain conceptual or preclinical, and to out knowledge there are no clinical reports of gene-editing or RNA-based therapy in CHI [18].

### 6.3. Collaborative Infrastructure

Equally important is the continued development of international collaborative research networks and patient registries. Given the rarity and marked genetic heterogeneity of CHI, multi-centre collaboration remains essential for establishing robust genotype–phenotype correlations, evaluating long-term outcomes and conducting adequately powered clinical trials [2,4,18].

Ultimately, the future of CHI lies in the integration of comprehensive genomic diagnostics with detailed clinical phenotyping and functional validation. Continued advances in genomics, functional biology, and precision therapeutics are expected not only to improve diagnostic yield but also to enable increasingly individualized management strategies that optimize outcomes while minimizing treatment-related morbidity [18].

## 7. Conclusions

CHI has evolved from a clinically defined disorder of persistent hypoglycaemia into a genetically and mechanistically diverse group of diseases that share a common phenotype of dysregulated insulin secretion. Advances in molecular genetics over the past three decades have transformed our understanding of β-cell physiology, revealing multiple molecular pathways that regulate β-cell function and insulin secretion. These discoveries have not only expanded the genetic landscape of CHI but have also provided fundamental insights into the mechanisms underlying normal β-cell function.

The clinical implications of these advances extend well beyond establishing a molecular diagnosis. Increasingly, identification of the causative genetic defect informs diagnosis, therapeutic selection, surgical planning, genetic counselling and long-term surveillance that enable transition from empirical management towards individualized, mechanism-based treatment strategies. Despite substantial progress, important challenges remain. A significant proportion of patients continue to lack a definitive molecular diagnosis. Early molecular diagnosis and the use of genotype to guide diazoxide trials, functional imaging, surgery and counselling are established practice; investigational agents remain unproven pending further trial evidence; and gene-based therapies remain proposals for future research.

CHI increasingly serves as a model for precision medicine in paediatric endocrinology. Future progress will depend upon continued multidisciplinary collaboration to integrate genomic discoveries into routine clinical practice. Ultimately, a pathway-based approach that combines comprehensive molecular diagnosis with individualized therapeutic strategies offers the greatest potential to improve clinical outcomes, minimize neurological morbidity, and optimize lifelong care for children affected by this complex and heterogenous disorder.

## Figures and Tables

**Table 1:** Clinically relevant genetic subtypes of congenital hyperinsulinism.

Gene	Protein	Mechanism	Inheritance	Characteristic Clinical Features	Diazoxide	Clinical Implications	Strength of Evidence
***ABCC8***	SUR1	K_ATP_ channel dysfunction due to impaired trafficking/gating	AR/AD/S	Most common form; severe neonatal CHI; diffuse or focal disease depending on inheritance	Variable	Paternal monoallelic variants raise the probability of focal disease → PET/CT before considering lesionectomy; biallelic disease usually diffuse	Established
***KCNJ11***	Kir6.2	KATP channel dysfunction	AR/AD/S	Similar to *ABCC8*; dominant variants often milder	Variable	Same surgical implications as *ABCC8*	Established
***GLUD1***	GDH	Loss of GTP inhibition	AD	HI/HA syndrome; protein-sensitive hypoglycaemia; persistent hyperammonaemia; neurological manifestations	Yes	Protein modification; surgery rarely required	Established
***GCK***	Glucokinase	Lower glucose threshold for insulin secretion	AD/S	Variable severity; neonatal or childhood onset; familial hypoglycaemia	Variable	Usually managed medically	Established
***HADH***	SCHAD	Loss of GDH inhibition	AR	Protein-sensitive hypoglycaemia without hyperammonaemia	Yes	Good prognosis; surgery rarely required	Established
***SLC16A1***	MCT1	Ectopic β-cell MCT1 expression	AD	Exercise-induced hypoglycaemia	Usually yes	Lifestyle modification; avoid unnecessary surgery	Established
***HNF4α/HNF1α***	Hepatocyte nuclear factors	Transcription factor defects	AD	Macrosomia; neonatal CHI; later MODY	Usually yes	Lifelong diabetes surveillance	Established
***HK1***	Hexokinase-1	Failure of β-cell gene silencing	AD	Fasting hypoglycaemia	Usually yes	Highlights importance of regulatory/non-coding variants	Established
***UCP2***	Uncoupling protein 2	Regulates mitochondrial proton transport and cellular energy metabolism; proposed to alter β-cell glucose oxidation and insulin secretion	AD	Post-prandial hypoglycaemia; hypoglycaemic seizures reported; usually appropriate for gestational age	Usually responsive	Rare and unconfirmed cause; pathogenic role remains debated and further validation is required	Disputed
***INSR***	Insulin receptor	Mechanism underlying hyperinsulinism remains uncertain	AD	Hyperinsulinism may evolve to severe insulin resistance and later diabetes; peri-pubertal androgen excess may occur	Variable	Consider when CHI coexists with insulin resistance or syndromic metabolic features	Limited

Strength of Evidence—Established: Pathogenicity supported by multiple unrelated families or cohorts with functional data; Limited: Few reported cases and/or incomplete functional validation; Disputed: Initial reports not confidently replicated in larger cohorts. AD: Autosomal dominant; AR: autosomal recessive; S: sporadic.

**Table 2:** Syndromic disorders associated with congenital hyperinsulinism and their distinguishing clinical features.

	Syndrome/Disorder	Gene/Chromosomal Abnormality	Mechanism of Hypoglycaemia	Major Associated Clinical Features	Clinical Significance	Strength of Evidence
**Overgrowth syndromes**	Beckwith–Wiedemann syndrome [64,65,66]	11p15 imprinting abnormalities	β-cell hyperplasia and dysregulated insulin secretion	Macroglossia, omphalocele, hemihyperplasia, embryonal tumour predisposition	Most common syndromic cause of neonatal hyperinsulinism; may require pancreatectomy	Established
	Perlman syndrome [67,68]	*DIS3L2*	Poorly defined	Prenatal overgrowth, nephroblastomatosis, Wilms tumour risk	Rare cause of neonatal hypoglycaemia	Limited
	Simpson–Golabi–Behmel syndrome [69,70]	*GPC3*	Poorly understood	Pre- and postnatal overgrowth, craniofacial anomalies, tumour predisposition	Hyperinsulinism reported in infancy	Limited
	Sotos syndrome [71,72,73]	*NSD1*	Unclear	Cerebral gigantism, developmental delay, macrocephaly	Occasional neonatal hypoglycaemia	Limited
	Weaver syndrome [74]	*EZH2*	Poorly defined	Generalized overgrowth, advanced bone age	Rare association with neonatal hypoglycaemia	Limited
	Costello syndrome [75,76,77,78,79]	*HRAS*	RAS/MAPK pathway dysregulation	Cardiomyopathy, developmental delay, tumour predisposition	Hyperinsulinism usually improves with age	Limited
**Monogenic developmental disorders**	CHARGE syndrome [80,81,82]	*CHD7*	Developmental dysregulation of endocrine pathways	Coloboma, heart defects, choanal atresia, growth retardation	Hyperinsulinism reported in isolated cases	Limited
	Coffin–Siris syndrome [83,84,85]	*ARID1B* and SWI/SNF complex genes	Poorly understood	Developmental delay, coarse facies, hypoplastic nails, obesity	Hyperinsulinism increasingly recognised	Limited
	*FOXA2* syndrome [54,55,86]	*FOXA2*	Abnormal pancreatic and pituitary development	Hyperinsulinism, hypopituitarism, craniofacial anomalies	Combined endocrine deficiency requires long-term follow-up	Established
	Rubinstein–Taybi syndrome [87,88,89,90]	*CREBBP*, *EP300*	Impaired transcriptional regulation of β-cell proliferation	Intellectual disability, broad thumbs, characteristic facies	Persistent hyperinsulinism increasingly recognised	Limited
	Schaaf–Yang syndrome [91]	*MAGEL2*	Unclear	Neonatal hypotonia, developmental delay, endocrine abnormalities	Hyperinsulinism reported in isolated patients	Limited
	Congenital central hypoventilation syndrome [92,93,94]	*PHOX2B*	Autonomic dysregulation	Central hypoventilation, autonomic dysfunction	Requires careful glucose monitoring	Limited
	MEHMO syndrome [95,96]	*EIF2S3*	Impaired β-cell function and pituitary development	Intellectual disability, epilepsy, hypogonadism, hypopituitarism	Variable glucose dysregulation from infancy to diabetes	Limited
	*DNAJC3* deficiency [97]	*DNAJC3*	Endoplasmic reticulum stress	Neurodevelopmental disorder, diabetes later in life	May initially present with insulin dysregulation	Limited
	*TRMT10A* deficiency [98,99]	*TRMT10A*	Abnormal β-cell survival	Microcephaly, intellectual disability, diabetes	Biphasic glucose phenotype reported	Limited
**Metabolic disorders**	PMM2-CDG [60,61,100]	*PMM2*	Defective glycosylation affecting β-cell function	Polycystic kidneys, congenital hepatic fibrosis	Distinctive syndromic hyperinsulinism	Established
	Tyrosinaemia type I [101,102,103]	*FAH*	Secondary β-cell dysfunction	Liver failure, renal Fanconi syndrome	Hyperinsulinism may resolve after metabolic treatment	Limited
**Channelopathies and membrane disorders**	Long QT syndrome [104]	*KCNQ1*	Altered *KCNQ1* channel activity affecting insulin secretion	Long QT interval, arrhythmias	Important cardiac association; avoid QT-prolonging drugs	Limited
	CACNA1D-related disorder [57,58]	*CACNA1D*	Gain-of-function calcium channel causing increased insulin exocytosis	Hyperaldosteronism, hypotonia, developmental delay	Illustrates calcium channel-mediated hyperinsulinism	Established
	CaV1.2-related disorder [59]	*CACNA1C*	Mixed gain/loss of calcium channel function	Cardiac manifestations	Rare metabolic cause	Limited
**Chromosomal syndromes**	Chromosome 9p deletion syndrome [63]	9p deletion	Developmental gene dosage effects	Developmental delay, dysmorphism	Rare chromosomal association	Limited
	16p11.2 deletion syndrome [105,106,107]	16p11.2 deletion	Unclear	Developmental delay, obesity, neurodevelopmental disorders	Hyperinsulinism increasingly recognised	Limited
	Trisomy 13 [108,109,110]	Trisomy 13	Poorly understood	Multiple congenital anomalies	Rare neonatal hyperinsulinism	Limited
	Trisomy 21 [111]	Trisomy 21	Multifactorial	Down syndrome	Increased referrals for CHI genetic testing; non-genetic contributors predominate	Limited
	Turner syndrome [112]	Monosomy X	Possible *KDM6A* haplo-insufficiency	Turner syndrome phenotype	Rare association with congenital hyperinsulinism	Limited
	Usher–hyperinsulinism syndrome [113,114]	Contiguous 11p deletion (*USH1C* region)	β-cell dysfunction	Deafness, enteropathy, renal tubulopathy, retinitis pigmentosa	May later progress to diabetes	Established

Strength of Evidence: Established—Association supported by multiple unrelated patients or cohorts with mechanistic or genetic data; Limited—Based on isolated case reports or small series, or on incompletely characterized mechanisms.

**Table 3:** Differential diagnosis of Hypoglycaemia without inappropriate Insulin secretion (CHI mimics).

Syndrome/Disorder	Gene/Chromosomal Abnormality	Mechanism of Hypoglycaemia	Major Associated Clinical Features	Clinical Significance
*PIK3CA*-related overgrowth spectrum (PROS) [115,116]	*PIK3CA*	PI3K pathway activation with enhanced insulin signalling	Segmental overgrowth, vascular malformations	Mosaic disease; hypoglycaemia may be overlooked
*AKT2*-related disorder [117,118,119]	*AKT2*	Constitutive insulin signalling causing glucose uptake independent of insulin	Hypoketotic hypoglycaemia without pancreatic insulin secretion, asymmetric overgrowth	Hypoglycaemia occurs despite suppressed insulin levels
*PGM1* deficiency [62]	*PGM1*	Abnormal glycogen metabolism and glycosylation	Cardiomyopathy, hepatopathy, endocrine abnormalities	Treatable metabolic disorder
Adenosine kinase deficiency [120]	*ADK*	Disordered hepatic energy metabolism	Liver dysfunction, hypermethioninemia, developmental delay	Rare metabolic cause of hypoglycaemia

**Table 4:** Established, investigational and proposed therapies for CHI.

Therapy	Mechanism	Target Patients	Evidence Level *
Diazoxide	K_ATP_ channel opener	Diazoxide-responsive CHI	Established (approved)
Octreotide, lanreotide	SSTR2/SSTR5 agonists	Diazoxide-unresponsive CHI	Established (off-label)
Glucagon infusion	Hepatic glucose release	Bridging therapy	Established (off-label)
Surgery (lesionectomy, near-total pancreatectomy)	Removal of hyperfunctioning tissue	Focal disease; medically unresponsive diffuse disease	Established
Avexitide	GLP-1 receptor antagonist	Diazoxide-unresponsive CHI	Investigational (early-phase)
Dasiglucagon	Stable glucagon analogue	Infants and children with CHI	Investigational (Two phase 3 trials; one missed its primary endpoint; not yet approved)
Ersodetug	Insulin receptor-modulating antibody	CHI despite standard care	Investigational (phase 3; primary endpoint not met)
Pasireotide; selective SSTR agonists	Broader or selective somatostatin receptor targeting	Diazoxide-unresponsive CHI	Investigational (case reports/early development)
Sirolimus	mTOR inhibitor	Severe unresponsive diffuse CHI	Off-label, use with caution
Gene editing, RNA-based therapy, KATP chaperones	Correction of the molecular defect	Genotype-specific	Preclinical/proposed

Evidence level *: Established, supported by consensus guidelines, replicated cohort data, or controlled trials, and in routine use (labelled “off-label” where the agent is not formally approved for CHI). Investigational, under evaluation in clinical studies or reported only in small series or case reports; not established for routine use. Preclinical/proposed, conceptual or laboratory-based strategies without clinical data. Regulatory and trial status reflects publicly available information at the time of the literature search (2026) and may change.

## Data Availability

No new data were created or analyzed in this study. Data sharing is not applicable to this article.

## Abbreviations

The following abbreviations are used in this manuscript:

*ABCC8*	ATP-binding cassette subfamily C member 8
AD	Autosomal dominant
*ADK*	Adenosine Kinase
ADP	Adenosine Diphosphate
*AKT2*	AKT serine/threonine kinase 2
AR	Autosomal recessive
*ARID1B*	AT-rich interactive domain 1B
ATP	Adenosine triphosphate
*CACNA1C*	Calcium voltage-gated channel subunit alpha1 C
*CACNA1D*	Calcium voltage-gated channel subunit alpha1 D
CDG	Congenital disorders of glycosylation
*CDKN1C*	Cyclin dependent kinase inhibitor 1C
*CHD7*	Chromodomain Helicase DNA binding protein 7
CHI	Congenital Hyperinsulinism
CGM	Continuous glucose monitoring
*CREBBP*	CREB binding protein
*DIS3L2*	DIS3 like exoribonuclease 2
*DNAJC3*	DnaJ heat shock protein family (Hsp40) member C3
*EIF2S3*	Eukaryotic translation initiation factor 2 subunit 3 (gamma)
*EP300*	E1A binding protein p300
*EZH2*	Enhancer of Zeste homolog 2
*FAH*	Fumarylacetoacetate hydrolase
*FOXA2*	Forkhead box A2
*GCK*	Glucokinase
GDH	Glutamate dehydrogenase
*GLUD1*	Glutamate dehydrogenase–1
GLP-1 receptor	Glucagon-like peptide-1 receptor
*GPC3*	Glypican protein coding 3
GTP	Guanosine triphosphate
*HADH*	3- hydroxyacyl-CoA dehydrogenase
HI/HA	Hyperinsulinism–Hyperammonaemia
*HNF4α*	Hepatocyte nuclear factor 4 alpha
*HNF1α*	Hepatocyte nuclear factor 1 alpha
*HK1*	Hexokinase 1
*HRAS*	HRas proto-oncogene, GTPase
*INSR*	Insulin receptor
K_ATP_	ATP-sensitive potassium channel
*KCNJ11*	Potassium inwardly rectifying channel subunit J member 11
*KCNQ1*	Potassium voltage-gated channel subfamily Q member 1
*KDM6A*	Lysine demethylase 6A
Kir6.2	Inwardly rectifying potassium channel 6.2
*KMT2D*	Lysine methyltransferase 2D
*MAFA*	MAF bZIP transcription factor A
*MAGEL2*	MAGE family member L2
MCT1	monocarboxylate transporter-1
MODY	maturity-onset diabetes of the young
mTOR	Mechanistic target of rapamycin
NGS	Next-generation sequencing
*NSD1*	Nuclear receptor binding SET domain protein 1
PET/CT	Positron emission tomography/computed tomography
*PGM1*	Phosphoglucomutase 1
*PMM2*	Phosphomannomutase 2
*PIK3CA*	Phosphatidylinositol-4,5-biphosphate 3-kinase catalytic subunit alpha
*PHOX2B*	Paired-like homeobox 2b
QT	QT interval
RAS/MAPK	Rat sarcoma/mitogen-activated protein kinase
SCHAD	short-chain 3-hydroxyacyl-CoA dehydrogenase
*SLC16A1*	Solute carrier family 16 member 1
SUR1	Sulfonylurea receptor 1
SSTR	Somatostatin receptor
SWI/SNF	SWI/SNF chromatin-remodelling complex
*TRMT10A*	tRNA methyltransferase 10A
*UCP2*	Uncoupling protein 2
*USH1C*	USH1 protein network component harmonin
^18^F-DOPA PET/CT	18F-fluoro-L-dihydroxyphenylalanine positron emission tomography/computed tomography
